# Validation and modification of existing mortality prediction models for lower gastrointestinal bleeding: a retrospective study

**DOI:** 10.1186/s12876-021-02037-4

**Published:** 2021-11-29

**Authors:** Hyun Seok Lee, Hee Seok Moon, In Sun Kwon, Hyun Yong Jeong, Byung Seok Lee, Seok Hyun Kim, Eaum-Seok Lee, Jae Kyu Sung, Sun Hyung Kang

**Affiliations:** 1 Division of Gastroenterology, Department of Internal Medicine, Chungnam National University Hospital, Chungnam National University School of Medicine, 282, Munhwa-ro, Juong-gu, Daejeon, 35015 Republic of Korea; 2 Clinical Trials Center, Chungnam National University, 282, Munhwa-ro, Juong-gu, Daejeon, 35015 Republic of Korea

**Keywords:** Lower gastrointestinal bleeding, hematochezia, Scoring system, Mortality prediction

## Abstract

**Background:**

Lower gastrointestinal bleeding (LGIB) often subsides without medical intervention; however, in some cases, the bleeding does not stop and the patient’s condition worsens. Therefore, predicting severe LGIB in advance can aid treatment. This study aimed to evaluate variables related to mortality from LGIB and propose a scoring system.

**Methods:**

In this retrospective study, we reviewed the medical records of patients who visited the emergency room with hematochezia between January 2016 and December 2020. Through regression analysis of comorbidities, medications, vital signs, laboratory investigations, and duration of hospital stay, variables related to LGIB-related mortality were evaluated. A scoring system was developed and the appropriateness with an area under the receiver operating characteristics curve (AUROC) was evaluated and compared with other existing models.

**Results:**

A total of 932 patients were hospitalized for LGIB. Variables associated with LGIB-related mortality were the presence of cancer, heart rate > 100 beats/min, blood urea nitrogen level ≥ 30 mg/dL, an international normalized ratio > 1.50, and albumin level ≤ 3.0 g/dL. The AUROCs of the models CNUH-4 and CNUH-5 were 0.890 (*p* < 0.001; cutoff, 2.5; 95% confidence interval, 0.0851–0.929) and 0.901 (*p* < 0.001; cutoff, 3.5; 95% confidence interval, 0.869–0.933), respectively.

**Conclusions:**

The model developed for predicting the risk of LGIB-related mortality is simple and easy to apply clinically. The AUROC of the model was better than that of the existing models.

## Background

Gastrointestinal bleeding (GIB) is anatomically classified based on the ligament of Treitz. Upper gastrointestinal bleeding (UGIB) is defined as bleeding above the ligament of Treitz, while lower gastrointestinal bleeding (LGIB) is defined as bleeding below the ligament of Treitz. Recently, with the introduction of small intestine endoscopy, small intestine bleeding is referred to as mid-gut bleeding, while colon and rectal bleeding below the ileocecal valve is called LGIB [1]. LGIB accounts for 20–40% of all GIB [1–3]. Compared with past data, the incidence of UGIB has reduced, whereas that of LGIB has increased. In the United States, the incidence of UGIB was 87 per 100,000 individuals in 1996 but decreased to 47 per 100,000 individuals in 2005. In contrast, the incidence of LGIB was 20 per 100,000 individuals in 1996 but increased to 33 per 100,000 individuals in 2005 [4, 5].

Various studies have evaluated the risk factors and mortality related to UGIB. Evaluation methods, such as AIMS65 [6] and Protetto Nazionale Emorragia Digestiva [7], which can predict UGIB-related mortality, and the Glasgow Blatchford Score (GBS) [8], which predicts the need for intervention after hospitalization, have been developed and applied in clinical practice. However, studies on LGIB are fewer than those on UGIB. This is because the incidence of LGIB is less than that of UGIB, and 80–85% of LGIB cases improve spontaneously without requiring hospitalization [9]. However, various studies are currently being undertaken to evaluate the mortality, severity, and risk factors of LGIB.

Compared with UGIB, no specific method has been established for evaluating the severity of LGIB. Hence, AIMS65, which is used for UGIB evaluation, is also used for LGIB evaluation. Recently, the age, blood tests, and comorbidities (ABC) score has been successfully developed for predicting LGIB-related mortality [10]. Thus, in this study, we aimed to evaluate variables related to mortality from LGIB and propose a scoring system. Additionally, this scoring system will be compared to AIMS65 and ABC.

## Methods

### Study design and population

In this retrospective study, all patient information was obtained through electronic medical records. Patients with hematochezia aged > 19 years who visited the emergency room (ER) of Chungnam National University Hospital from January 1, 2016 to December 31, 2020 were included. Among these, patients discharged from the hospital on the same day after treatment in the ER were excluded and only patients who received inpatient treatment were included. Causes of LGIB were classified based on investigational results such as duodenoscopy, colonoscopy, angiography, and abdominal hemorrhagic computed tomography (CT) among hospitalized patients.

### Selection of predictive variables

Using previous studies as a reference, variables that are expected to be associated with LGIB-related mortality were searched using electronic medical records. Basic patient-related information included age, sex, comorbidities (cancer, heart failure [HF], renal failure [RF], liver cirrhosis [LC], chronic obstructive pulmonary disease [COPD]), and medication history (anticoagulant, antiplatelet, and non-steroidal anti-inflammatory drugs [NSAIDs]). Data on vital signs and blood test (hemoglobin level, hematocrit percentage, platelet count, albumin level, blood urea nitrogen [BUN] level, creatinine level, and international normalized ratio [INR]) at the ER, hospitalization duration, intensive care unit treatment duration, and death were also collected. The American Society of Anesthesiologists (ASA) scores are required to calculate the ABC score.

### Primary outcome and definitions

LGIB was defined as colon and rectal bleeding below the ileocecal valve confirmed through duodenoscopy, colonoscopy, angiography, and hemorrhagic CT. The primary outcome was to investigate variables related to all-cause mortality of patients hospitalized with LGIB. The secondary outcome was to evaluate the area under the receiver operating characteristics curve (AUROC) values of the primary outcomes. The newly introduced AUROC in this study will be called CNUH, which stands for Chungnam National University Hospital. The CNUH was compared with those of existing models (ABC and AIMS65).

### Statistical analyses

For continuous variables, the mean value was used when the variable values were relatively evenly distributed, and the median value was used otherwise. Categorical variables are represented as numbers (percentages). Univariate logistic regression analysis was used to investigate variables associated with all-cause mortality, and the Hosmer–Lemeshow test was conducted to determine the suitability of the results. Multivariable logistic regression analysis was used to determine the actual role of each factor by correcting the influences of the variables with meaningful results. While selecting statistically meaningful variables in the univariable logistic regression analysis, to reduce the error of selecting confounding and suppressor variables, the p-value was set at < 0.1. For the secondary outcome, four to five variables most related to all-cause mortality in the primary outcome were selected, and then each AUROC value was compared. SPSS version 22 (IBM Corp., Armonk, NY, USA) was used for the statistical analyses.

## Results

### Patient characteristics

**Fig. 1:**
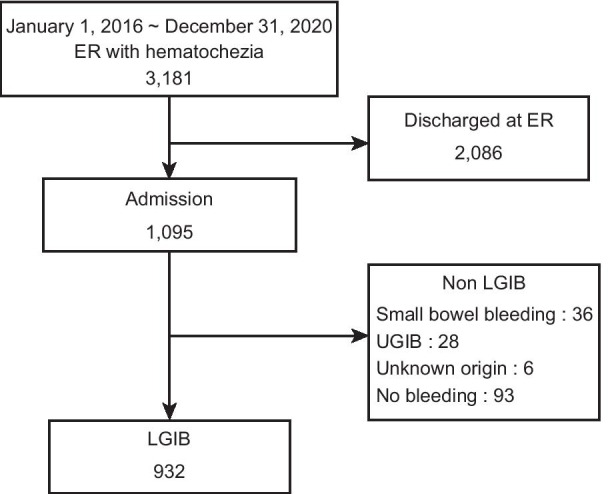
Study flow chart

From January 2016 to December 2020, 3181 patients with hematochezia aged ≥ 19 years older were admitted to the ER. Of them, 2086 were discharged on the same day after treatment and 1095 were hospitalized for LGIB. Finally, 932 patients were diagnosed with LGIB (Fig. 1).

Among patients who underwent inpatient treatment, 57.3% were men, and 42.7% were women. The median age was 68 years. Underlying comorbidities included cancer 18.8%, HF 12.0%, RF 12.4%), COPD 2.9%, and LC 10.8%. ASA scores were as follows: 1 point 58.9%; 2 points 13.8%; and 3 points 27.2%. In the ER, 1045 patients were alert and 50 had altered mental status. During hospitalization, the concomitant medications were antiplatelet agents 18.8%; anticoagulants 11.1%; and NSAIDs 27.9%. The median length of hospital stay was 6 days. Moreover, 51 patients received intensive care treatment and the median duration of intensive care unit treatment was 3 days (Table 1).

**Table 1:** Baseline characteristics of the patients

Parameters	Results	
Sex (%)	Male: 627 (57.3)	Female: 468 (42.7)
Age, median (range)	68 (19–94)	
Comorbidity (%)	Cancer	206 (18.8)
	Heart failure	131 (12.0)
	Renal failure	136 (12.4)
	Chronic pulmonary obstructive disease	32 (2.9)
	Liver cirrhosis	118 (10.8)
ASA score	1	645 (58.9)
	2	152 (13.8)
	3	298 (27.2)
Mental status	Alert	1045 (95.4)
	Altered	50 (4.5)
Medication (%)	Antiplatelet	206 (18.8)
	Anticoagulant	122 (11.1)
	Non-steroidal anti-inflammatory drugs	306 (27.9)
Hospitalization	Day, median (range)	6.00 (1–203)
	*Intensive care unit*	
	n (%)	51 (4.65)
	Day, median (range)	3.00 (1–26)
Mortality (%)	All-cause mortality	40 (3.7)
	Bleeding-related mortality	23 (2.1)
	Non-bleeding-related mortality	17 (1.6)

ASA, American Society of Anesthesiologists

### Vital signs, laboratory results, and mortality

Among patients who underwent inpatient treatment for LGIB, 40 (3.7%) died; among these, 23 (2.1%) died from LGIB and 17 (1.6%) died from causes other than LGIB (Table 1). The median heart rate was 100 beats/min, the median systolic blood pressure was 100 mmHg, and the median diastolic blood pressure was 53 mmHg (Table 2). The mean hemoglobin level and hematocrit percentage were 9.24 g/dL and 27.22%, respectively. The median platelet count, BUN level, creatinine level, albumin level, and INR were 161,000/µL, 22.3 mg/dL, 0.95 mg/dL, 3.0 g/dL, and 1.12, respectively (Table 2).

**Table 2:** Vital signs and laboratory test results at the emergency room

Parameters	Results
Vital signs (median)	
Heart rate (beat/min)	100
Systolic blood pressure (mmHg)	100
Diastolic blood pressure (mmHg)	53
Laboratory test results	
Hemoglobin, mean(g/dL)	9.24
Hematocrit, mean (%)	27.22
Platelet, median (µL)	161,000
Blood urea nitrogen, median (mg/dL)	22.3
Creatinine, median (mg/dL)	0.95
Albumin, median (g/dL)	3.0
International normalized ratio, median	1.12

### Endoscopic results

Of 1095 patients admitted for LGIB, 850 underwent endoscopy. Among these, 144 patients underwent endoscopic hemostasis, 24 underwent transarterial embolization and one underwent both procedures (Table 3). In endoscopy, the most common cause of LGIB was ischemic colitis 16.3%, followed by diverticular bleeding 16.0%, ulcer bleeding 6.8%, cancer bleeding 6.2%, and post-polypectomy bleeding 5.6%. The cause was not found in 6 patinets complaining hematochezia and 93 patients showed no sign of bleeding on endoscopy(Table 4).

**Table 3:** The proportion of patients who underwent endoscopy and intervention

Intervention	Results
Endoscopic exam, n (%)	850 (77.6)
Intervention, n (%)	
Endoscopic bleeding control	144 (13.2)
Embolization	24 (2.2)
Both of the above	1 (0.1)

**Table 4:** Endoscopic results

Endoscopic findings	n (%)
Ischemic colitis	178 (16.3)
Diverticular bleeding	175 (16.0)
Ulcer bleeding	75 (6.8)
Cancer bleeding	68 (6.2)
Post polypectomy bleeding	61 (5.6)
Ulcerative colitis	34 (3.1)
Colitis	30 (2.7)
Polyp	27 (2.5)
Adenoma	13 (1.2)
Varix bleeding	8 (0.7)
Angiodysplasia	6 (0.5)
Pseudomembranous colitis	4 (0.4)
Arteriovenous malformation	3 (0.3)
Crohn’s disease	3 (0.3)
Subepithelial tumor	3 (0.3)
International normalized ratio prolongation	1 (0.1)

### Correlation with all-cause mortality

In the univariate analysis, the following factors were associated with all-cause mortality: age ≥ 75 years, presence of cancer, heart rate > 100 beats/min, systolic blood pressure < 100 mmHg, diastolic blood pressure < 60 mmHg, hemoglobin ≤ 10.0 mg/dL, hematocrit ≤ 30.0%, platelet ≤ 100,000/µL, BUN ≥ 30 mg/dL, creatinine lev > 1.5 mg/dL, albumin ≤ 3.0 g/dL, and INR > 1.50 (Table 5). The P-value of the Hosmer–Lemeshow test conducted to determine suitability was 0.996.

**Table 5:** Logistic regression analyses results for risk factors of mortality from lower gastrointestinal bleeding

	Univariable logistic regression analysis			Multivariable logistic regression analysis		
	OR	*p*-value	95% CI	OR	*p*-value	95% CI
Aged ≥ 75 years	2.110	0.021	1.120–3.977	1.203	0.641	0.553–2.619
Male sex	0.845	0.602	0.449–1.591			
Cancer	2.713	0.003	1.403–5.246	2.081	0.071	0.939–4.609
COPD	0.915	0.931	0.121–6.897			
Renal failure	1.559	0.298	0.675–3.601			
Heart failure	1.373	0.485	0.564–3.339			
Liver cirrhosis	2.172	0.057	0.976–4.836			
Antiplatelet	0.758	0.537	0.314–1.830			
Anticoagulant	1.144	0.783	0.439–2.979			
NSAIDs	0.992	0.983	0.489–2.013			
Heart rate (> 100 beats/min)	39.198	0.000	5.333–288.086	13.134	0.014	1.677–102.880
SBP (< 100 mmHg)	9.384	0.000	3.279–26.854	2.477	0.188	0.642–9.560
DBP (< 60 mmHg)	6.783	0.009	1.612–28.538	0.942	0.950	0.149–5.958
Hemoglobin (≤ 10.0 mg/dL)	2.712	0.013	1.238–5.945	1.023	0.982	0.146–7.176
Hematocrit (≤ 30.0%)	3.905	0.007	1.356–7.063	0.296	0.264	0.035–2.501
Platelet (≤ 100,000/uL)	3.809	0.000	2.013–7.206	1.103	0.811	0.494–2.463
BUN (≥ 30 mg/dL)	11.314	0.000	4.950–25.860	3.760	0.016	1.279–11.051
Creatinine (> 1.5 mg/dL)	4.429	0.000	2.336–8.395	1.070	0.881	0.442–2.590
Albumin (≤ 3.0 g/dL)	37.509	0.000	5.134–274.040	7.360	0.072	0.839–64.574
INR (> 1.50)	10.321	0.000	5.310–20.061	3.258	0.005	1.424–7.454

OR, odds ratio; CI, confidence interval; COPD, chronic obstructive pulmonary disease; NSAIDs, non-steroidal anti-inflammatory drugs; SBP, systolic blood pressure; DBP, diastolic blood pressure; BUN, blood urea nitrogen; INR, international normalized ratio. Hosmer–Lemeshow test *p*-value 0.996(χ^2^1.224; df 8)

In the multivariable logistic regression analysis, significant (*p* < 0.05) results were obtained with the following variables: heart rate > 100 beats/min, BUN ≥ 30 mg/dL and INR > 1.50 (Table 5).

### CNUH model for the prediction of mortality

To determine the predictability of all-cause mortality from the variables, the AUROC was evaluated. Three variables with meaningful results in multivariable analysis were selected. In addition, presence of cancer and albumin ≤ 3.0 g/dL, which were not statistically meaningful but were thought to have a relative effect on all-cause mortality, were selected.

Two models were compared. The first one was called a CNUH-4 model that incorporated the following variables: cancer, heart rate > 100 beats/min, BUN ≥ 30 mg/dL, and INR > 1.50. The AUROC was drawn by assigning points from 0 to 4. The second model (CNUH-5) was created by adding an albumin ≤ 3.0 g/dL to the CNUH-4 model. The AUROC was drawn by assigning points from 0 to 5.

**Fig. 2:**
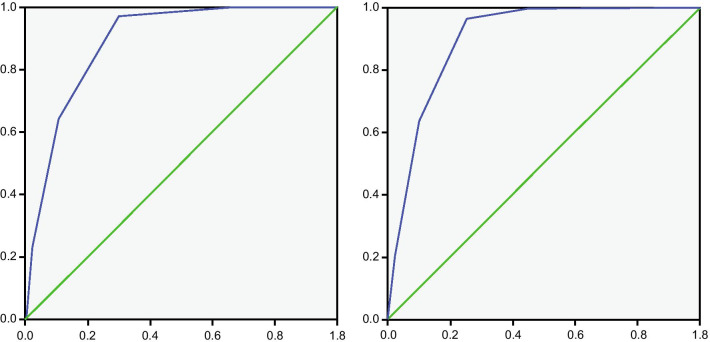
Comparison of the area under the receiver operating characteristics curve (AUROC) between CNUH-4 and CNUH-5. The AUROC of CNUH-4 (left) was 0.890 (*p* < 0.001; cutoff, 2.5; 95% CI, 0.0851–0.929). The AUROC of CNUH-5 (right) was 0.901 (*p* = 0.000; cutoff, 3.5; 95% CI, 0.869–0.933). x = 1 − sensitivity, y = sensitivity

The score distribution in the CNUH-4 model was as follows: 0 points, 310 (33.5%) patients; 1 point, 318 (34.3%) patients; 2 points, 183 (19.8%) patients; 3 points, 91 (9.8%) patients; and 4 points, 24 (2.6%) patients. The AUROC was 0.890 (*p* < 0.001; cutoff, 2.5; 95% CI, 0.0851–0.929) (Fig. 2). The score distribution in the CNUH-4 model was as follows: 0 points, 223 (24.1%) patients; 1 point, 254 (27.4%) patients; 2 points, 190 (20.5%) patients; 3 points, 148 (16.0%) patients; 4 points, 88 (9.5%) patients; and 5 points, 23 (2.5%) patients. The AUROC was 0.901 (*p* < 0.001; cutoff, 3.5; 95% CI, 0.869–0.933), higher than that of CNUH-4 (Fig. 2).

**Fig. 3:**
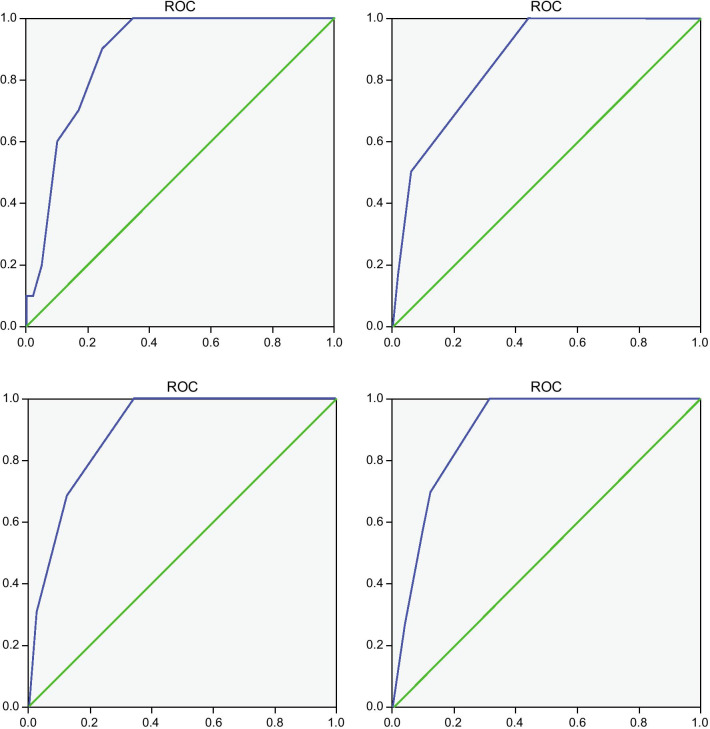
Comparison of various models using patient data in 2020. The area under the receiver operating characteristics curve (AUROC) of ABC (left upper) was 0.881 (*p* < 0.001; 95% CI, 0.817–0.945), the AUROC of AIMS65 (right upper) was 0.861 (*p* < 0.001; 95% CI, 0.771–0.951), the AUROC of CNHU-4 (left lower) was 0.893 (*p* < 0.001; 95% CI, 0.826–0.960), and the AUC of CNUH-5 (right lower) was 0.896 (*p* < 0.001; 95% CI, 0.836–0.956). x = 1 − sensitivity, y = sensitivity

The results of CNUH-4 and CNUH-5 were compared with those of ABC and AIMS65 using data from the Chungnam National University Hospital in 2020. The AUROC of ABC was 0.881 (*p* < 0.001; 95% CI, 0.817–0.945), the AUROC of AIMS65 was 0.861 (*p* < 0.001; 95% CI, 0.771–0.951), the AUROC of CNHU-4 was 0.893 (*p* < 0.001; 95% CI, 0.826–0.960), and the AUROC of CNUH-5 was 0.896 (*p* < 0.001; 95% CI, 0.836–0.956). When compared with data in 2020, the AUROC of CNUH-5 was the highest (Fig. 3).

## Discussion

Several studies have introduced indicators to predict the severity and mortality of UGIB [6–8]. Similar to UGIB, a scoring system that can predict the prognosis of LGIB using various predictors at the time of ER visit will be useful. Although the prognosis of LGIB is better than that of UGIB, it is very important to develop a scoring system to assess the mortality risk in advance. Therefore, we re-evaluated the risk factors using the existing scoring system, including only patients with LGIB and undergoing inpatient treatment, and developed a new scoring system for clinical use.

Prior to this study, other studies have evaluated the mortality and severity of LGIB. In these studies, heart rate > 100 beats/min, systolic blood pressure < 115 mmHg, aspirin use, ≥ 2 comorbidities [11], hematocrit ≤ 35.0%, systolic blood pressure < 100 mmHg or heart rate > 100/min [12], transfusion, re-bleeding, 20% reduction of hematocrit [13], prothrombin time > 1.2-times the control, erratic mental status, unstable comorbidities [14], age ≥ 75 years, creatinine level > 150 µmol/L, and albumin level ≤ 30 g/L [9] were found to be associated with LGIB-induced severe morbidity and mortality.

Similar to previous studies, our univariable logistic regression analysis showed that specific vital signs, age, and laboratory test results were associated with LGIB-induced mortality. However, in the multivariable logistic regression analysis, variables associated with all-cause mortality were the presence of cancer, heart rate > 100 beats/min, BUN ≥ 30 mg/dL, albumin level ≤ 3.0 g/dL, and INR > 1.50. When comparing the two models, CNUH-5 had a higher AUROC value than CNUH-4 (0.901 vs. 0.890).

Two previous validation and derivation studies on LGIB severity have reported that the AUROC was 0.754 for the validation study and 0.761 for the derivation study [11, 15]. In a study comparing the relationship between eight variables and 30-day mortality, the AUROC was 0.72 [16]. In a study that performed external validation with the existing NOBLADS score, the in-hospital mortality rate was > 5 points (AUROC, 0.83) [17]. Compared with these studies, the AUROC of the CNUH model was found to be higher.

Few studies have evaluated the prognosis of LGIB. AIMS65, GBS, and Oakland scores have been found to be useful in assessing LGIB, and AIMS65 is known to be highly correlated with LGIB-related mortality [14, 18]. In this study, we compared AIMS65, which has the highest mortality prediction ability, with the recently developed ABC. Albumin level, INR, mental status, systolic blood pressure, and age are required to calculate the AIMS65 score, while age, BUN, albumin level, creatinine level, mental status, LC, and malignancy are required to calculate the ABC score. However, the CNUH model does not include mental status, ASA score, and age; thus, a direct comparison was impossible. Therefore, we used the CNUH model as the comparator and validated the ABC and AIMS65 models.

The AUROCs of ABC, AIMS65, CNUH-4, and CNUH-5 with patient data in 2020 were 0.881, 0.861, 0.893, and 0.896, respectively. A slight difference was observed between the AUROCs of CNUH-4 and CNUH-5 when all enrolled patients were included. It is meaningful that the model presented in this study had a relatively higher AUROC than the two existing ones, although the patient’s information or score calculation method was simpler with the CNUH model.

This study has some limitations. In this retrospective study, all information was obtained from electronic medical records. This method does not provide adequate information on the change of conditions from the time of admission to death or discharge. LGIB often improves spontaneously and has a low mortality rate [19–22]. Because of the low all-cause mortality rate, the OR values of some variables, such as heart rate, BUN, albumin, and INR, might be overestimated. In addition, there might be a selection bias in targeting only patients hospitalized for LGIB. However, the scoring system was exclusively developed for patients who visited the hospital with hematochezia and in whom LGIB was clearly identified. Finally, this study was based on a single tertiary-care institution; our results need to be validated in other settings in a larger cohort.

## Conclusions

We developed a model for predicting the risk of LGIB-related mortality, and five variables related to all-cause mortality were identified. Our model is simpler than the existing model. thus, it can be quickly applied when evaluating patients in the ER. Further studies are required to validate our results in a larger cohort.

## Data Availability

The datasets used and/or analyzed during the current study are available from the corresponding author on reasonable request.

## Abbreviations

ABC: Age, blood tests, and comorbidities
ASA: American Society of Anesthesiologists
AUROC: Area under the receiver operating characteristics curve
BUN: Blood urea nitrogen
CI: Confidence interval
COPD: Chronic obstructive pulmonary disease
CT: Computed tomography
ER: Emergency room
GBS: Glasgow Blatchford Score
HF: Heart failure
INR: International normalized ratio
LC: Liver cirrhosis
LGIB: Lower gastrointestinal bleeding
NSAIDs: Non-steroidal anti-inflammatory drugs
OR: Odds ratio
RF: Renal failure
UGIB: Upper gastrointestinal bleeding

## References

[1] ASGE Standards of Practice Committee; Pasha SF, Shergill A, Acosta RD, Chandrasekhara V, Chathadi KV, et al. The role of endoscopy in the patient with lower GI bleeding. Gastrointest Endosc. 2014;79:875–85. 10.1016/j.gie.2013.10.039 24703084

[2] Strate LL, Gralnek IM (2016). ACG clinical guideline: management of patients with acute lower gastrointestinal bleeding. Am J Gastroenterol.

[3] Gralnek IM, Neeman Z, Strate LL (2017). Acute lower gastrointestinal bleeding. N Engl J Med.

[4] Lanas A, García-Rodríguez LA, Polo-Tomás M, Ponce M, Alonso-Abreu I, Perez-Aisa MA (2009). Time trends and impact of upper and lower gastrointestinal bleeding and perforation in clinical practice. Am J Gastroenterol.

[5] El-Tawil AM (2012). Trends on gastrointestinal bleeding and mortality: where are we standing?. World J Gastroenterol.

[6] Saltzman JR, Tabak YP, Hyett BH, Sun X, Travis AC, Johannes RS (2011). A simple risk score accurately predicts in-hospital mortality, length of stay, and cost in acute upper GI bleeding. Gastrointest Endosc.

[7] Marmo R, Koch M, Cipolletta L, Capurso L, Grossi E, Cestari R (2010). Predicting mortality in non-variceal upper gastrointestinal bleeders: validation of the Italian PNED score and prospective comparison with the rockall score. Am J Gastroenterol.

[8] Blatchford O, Murray WR, Blatchford M (2000). A risk score to predict need for treatment for upper-gastrointestinal haemorrhage. Lancet.

[9] Farrell JJ, Friedman LS (2005). Review article: the management of lower gastrointestinal bleeding. Aliment Pharmacol Ther.

[10] Laursen SB, Oakland K, Laine L, Bieber V, Marmo R, Redondo-Cerezo E (2021). ABC score: a new risk score that accurately predicts mortality in acute upper and lower gastrointestinal bleeding: an international multicentre study. Gut.

[11] Strate LL, Orav EJ, Syngal S (2003). Early predictors of severity in acute lower intestinal tract bleeding. Arch Intern Med.

[12] Velayos FS, Williamson A, Sousa KH, Lung E, Bostrom A, Weber EJ (2004). Early predictors of severe lower gastrointestinal bleeding and adverse outcomes: a prospective study. Clin Gastroenterol Hepatol.

[13] Newman J, Fitzgerald JE, Gupta S, von Roon AC, Sigurdsson HH, Allen-Mersh TG (2012). Outcome predictors in acute surgical admissions for lower gastrointestinal bleeding. Colorectal Dis.

[14] Kollef MH, O'Brien JD, Zuckerman GR, Shannon W (1997). BLEED: a classification tool to predict outcomes in patients with acute upper and lower gastrointestinal hemorrhage. Crit Care Med.

[15] Strate LL, Saltzman JR, Ookubo R, Mutinga ML, Syngal S (2005). Validation of a clinical prediction rule for severe acute lower intestinal bleeding. Am J Gastroenterol.

[16] Sengupta N, Tapper EB (2017). Derivation and internal validation of a clinical prediction tool for 30-day mortality in lower gastrointestinal bleeding. Am J Med.

[17] Aoki T, Yamada A, Nagata N, Niikura R, Hirata Y, Koike K (2018). External validation of the NOBLADS score, a risk scoring system for severe acute lower gastrointestinal bleeding. PLoS ONE.

[18] Oakland K, Jairath V, Uberoi R, Guy R, Ayaru L, Mortensen N (2017). Derivation and validation of a novel risk score for safe discharge after acute lower gastrointestinal bleeding: a modelling study. Lancet Gastroenterol Hepatol.

[19] Angtuaco TL, Reddy SK, Drapkin S, Harrell LE, Howden CW (2001). The utility of urgent colonoscopy in the evaluation of acute lower gastrointestinal tract bleeding: a 2-year experience from a single center. Am J Gastroenterol.

[20] Browder W, Cerise EJ, Litwin MS (1986). Impact of emergency angiography in massive lower gastrointestinal bleeding. Ann Surg.

[21] Chaudhry V, Hyser MJ, Gracias VH, Gau FC (1998). Colonoscopy: the initial test for acute lower gastrointestinal bleeding. Am Surg.

[22] Longstreth GF (1997). Epidemiology and outcome of patients hospitalized with acute lower gastrointestinal hemorrhage: a population-based study. Am J Gastroenterol.

